# Safety Profile of a Multi-Antigenic DNA Vaccine Against Hepatitis C Virus

**DOI:** 10.3390/vaccines8010053

**Published:** 2020-01-29

**Authors:** Jason Gummow, Makutiro G. Masavuli, Zelalem A. Mekonnen, Yanrui Li, Danushka K. Wijesundara, Ashish C. Shrestha, Ilia Voskoboinik, Eric J. Gowans, Branka Grubor-Bauk

**Affiliations:** 1 Virology Laboratory, Discipline of Surgery, The University of Adelaide and Basil Hetzel Institute for Translational Health Research, Adelaide 5011, Australia; 2 Gene Silencing and Expression Laboratory, Robinson Research Institute, The University of Adelaide, Adelaide 5000, Australia; 3 International Centre for Allied Health Evidence and Sansom Institute for Health Research, University of South Australia, Adelaide 5000, Australia; 4 School of Chemistry and Molecular Biosciences, University of Queensland, Brisbane 4072, Australia; 5 Killer Cell Biology Laboratory, Cancer Immunology Research, Peter MacCallum Cancer Centre, Victoria 3000, Australia

**Keywords:** cell death, DNA vaccine, Hepatitis C Virus, immune breadth, Pre-clinical, pathology, perforin, toxicology

## Abstract

Despite direct acting antivirals (DAAs) curing >95% of individuals infected with hepatitis C (HCV), in order to achieve the World Health Organization HCV Global Elimination Goals by 2030 there are still major challenges that need to be overcome. DAAs alone are unlikely to eliminate HCV in the absence of a vaccine that can limit viral transmission. Consequently, a prophylactic HCV vaccine is necessary to relieve the worldwide burden of HCV disease. DNA vaccines are a promising vaccine platform due to their commercial viability and ability to elicit robust T-cell-mediated immunity (CMI). We have developed a novel cytolytic DNA vaccine that encodes non-structural HCV proteins and a truncated mouse perforin (PRF), which is more immunogenic than the respective canonical DNA vaccine lacking PRF. Initially we assessed the ability of the HCV pNS3-PRF and pNS4/5-PRF DNA vaccines to elicit robust long-term CMI without any adverse side-effects in mice. Interferon-γ (IFN-γ) enzyme-linked immunosorbent spot (ELISpot) assay was used to evaluate CMI against NS3, NS4 and NS5B in a dose-dependent manner. This analysis showed a dose-dependent bell-curve of HCV-specific responses in vaccinated animals. We then thoroughly examined the effects associated with reactogenicity of cytolytic DNA vaccination with the multi-antigenic HCV DNA vaccine (pNS3/4/5B). Hematological, biochemical and histological studies were performed in male Sprague Dawley rats with a relative vaccine dose 10–20-fold higher than the proposed dose in Phase I clinical studies. The vaccine was well tolerated, and no toxicity was observed. Thus, the cytolytic multi-antigenic DNA vaccine is safe and elicits broad memory CMI.

## 1. Introduction

HCV is one of the most prevalent blood-borne viruses, with 71 million people infected and an estimated 1.75 million new cases worldwide in 2015 [1,2]. The high prevalence is attributed to the ability of HCV to cause persistent infection in 70–80% of cases, resulting in a large reservoir of infectious individuals [3]. Over time, persistent HCV infection results in liver fibrosis, steatosis and cirrhosis and may progress to hepatocellular carcinoma [4,5]. Therefore, HCV infection remains a considerable global public health burden [6].

Current interferon (IFN)-free, direct-acting antiviral (DAA) therapy cures >95% of treated individuals [7,8] with fewer side effects than IFN-based treatment [9,10]. However, only 20% of infected individuals worldwide are diagnosed and the cost of DAAs is unaffordable for patients in low- and middle-income countries. Importantly, successful DAA treatment does not prevent re-infection [11,12].

Thus, despite the effectiveness of DAA therapy, the ability of HCV to cause asymptomatic infection that may be unrecognised for a considerable period of time, and the cost of therapy demonstrates a clear need for a more practical option. In addition, only 20% of infected individuals are diagnosed [13], and access to DAA therapy for individuals in developing countries with the greatest need is limited. Thus, these individuals are likely to remain untreated and represent a reservoir for continuing transmission. Thus, DAAs alone are unlikely to eliminate HCV in the absence of a vaccine that can limit viral transmission. Consequently, an effective prophylactic vaccine is necessary to reduce the burden of HCV infection.

As HCV vaccine candidates have typically attempted to induce neutralising antibodies (NAb), but have failed to elicit protection, current dogma suggests that an HCV vaccine should be aimed at eliciting cell-mediated immunity (CMI), as well as NAb [14]. DNA-based vaccines elicit CMI as the expressed antigens are typically intracellular, making this technology suitable against intracellular pathogens. Immunisation with DNA vaccines results in expression of the natural form of the immunogen, such as transmembrane proteins, which would otherwise be truncated in peptide- or protein-based vaccines [15]; thus allowing comprehensive targeting of the antigen [16,17,18]. It presents an attractive vaccine technology due to its economic viability and shelf-life [16,19,20,21], as, unlike more conventional vaccines, DNA vaccines can be prepared quickly and cheaply, and a cold chain is not required [16,17,18]. Furthermore, DNA vaccines can be easily manipulated to encode a more immunogenic version of the native protein and can elicit CMI and/or humoral responses [22]. Although, historically, DNA vaccines have not been effective in large animals and humans, recent studies described therapeutic DNA vaccination against human papillomavirus (HPV) which resulted in histological regression and/or eliminated persistent HPV infection and HPV-related cervical lesions [23,24].

To date no DNA vaccine has resulted in autoimmunity and there is no observed risk of integration into the host genome [25].

Any successful vaccine aimed at eliciting a strong CMI must target dendritic cells (DC) either directly or indirectly via antigen cross-presentation to naïve T cells [26,27]. A number of studies in our laboratory have shown that a cytolytic DNA vaccine, which expresses C-terminal truncated mouse perforin, induces necrosis in vaccine-targeted cells, and is more effective than a canonical DNA vaccine, as it increases anti-viral effector CD8+ T cell immunity [28,29,30,31,32,33]. Previous studies have reported that this C-terminal truncated PRF (PRF-12) fails to be exported from the endoplasmic reticulum and is highly toxic to the host cell [34,35]. The concentration of Ca2+ in the endoplasmic reticulum activates PRF-12 to form pores and, as a consequence activates cell death pathways involved in regulated necrosis in PRF-transfected cells (reviewed by [36]).We have shown that this cytolytic DNA vaccine activates DC more efficiently to present vaccine-encoded antigens to CD8+ T cells *in vivo,* compared to canonical DNA vaccination [32]. Furthermore, a multi-antigenic cytolytic HCV vaccine encoding non-structural (NS) proteins NS3/4A/4B/5B, described in one of our studies, increased the breadth of the T cell responses to each of the encoded antigens in a polyprotein immunogen, without compromising the immunogenicity of the individual antigens [31]. More recently, we have demonstrated that a multi-genotypic DNA cocktail vaccine encoding gt1b and gt3a NS5B proteins induced higher CMI responses to gt1b and gt3a NS5B proteins compared to a DNA vaccine encoding a global consensus sequence [37], while a multi-antigenic DNA vaccine cocktail encoding gt1b and gt3a NS3, NS4, and NS5B proteins was significantly more effective at inducing responses to NS3 and NS5B than vaccination with a vaccine encoding the individual genotypes [37]. An important step to progress any promising vaccine candidate into clinical trials is to determine its safety profile. Thus, the aim of this study was to determine if the cytolytic vaccine resulted in any untoward side effects and to examine the cell-mediated immune responses in a dose-dependent manner.

## 2. Materials and Methods

### 2.1. Vaccines

**Figure 1:**
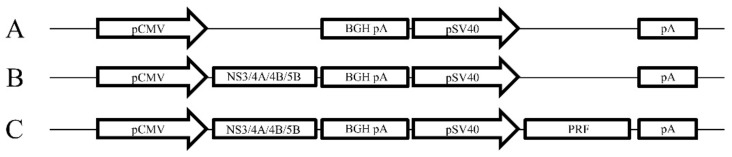
Schematic map of the vaccine constructs. (A) pVAX empty construct, (B) pVAX encoding NS3, 4A, 4B and 5B downstream of the CMV promoter (pNS3/4A/4B/5B) and (C) pNS3/4A/4B/5B encoding Perforin (PRF) downstream of the SV40 promoter (pNS3/4A/4B/5B-PRF).

The DNA plasmids were constructed in pVAX (Life Technologies) as described previously [31] (Figure 1). Codon-optimised genes (GeneArt, Regensburg, Germany) encoding the HCV proteins NS3, NS4A, a truncated form of NS4B with aa 1 to 84 deleted and NS5B from HCV genotype 3a (gt3a) (GenBank accession number AF046866) were inserted downstream of the cytomegalovirus (CMV) promoter (Figure 1). The simian virus 40 (SV40) promoter and a poly(A) sequence were also inserted to control expression of the cytolytic protein, perforin (PRF), lacking the final 12 residues at the C terminus [31,34] (Figure 1). All DNA vaccines were purified using the endotoxin-free Mega kit (Qiagen, Doncaster, Victoria, Australia).

### 2.2. Animals

All experiments followed the Australian code for the care and use of animals for scientific purposes and were approved by the The University of Adelaide and South Australian Pathology Animal Ethics Committees. Female C57BL/6 mice and male Sprague Dawley rats were bred in specific pathogen-free conditions and housed at The Queen Elizabeth Hospital animal facility in PC2 conditions.

### 2.3. Vaccination Studies

The mice were aged 6−8 weeks and weighed ~18 ± 1 grams at the start of the experiments. All interventions were performed under isoflurane anaesthesia. Mice received three or four doses (as indicated) of 50 µg of endotoxin-free DNA (25 µg/ear) or PBS injected into the ear pinnae (intradermal (ID) injection) at two week intervals as described previously [28,29,38,39]. Fourteen and 144 days after the final vaccination, the mice were culled, and splenocytes were prepared as described previously [30,31,40].

### 2.4. Toxicology and Histopathology

Three groups of 10, six week old male Sprague Dawley rats were injected via the ID route in the intrascapular region with 500 μL of medical grade saline or endotoxin-free DNA vaccine dissolved in 500 μL medical grade saline. The rats were immunised with 150 μg DNA on Day 0 followed by 450 μg DNA on Days 5 and 15, then euthanised and necropsies performed on Day 23. Tissues from the injection site, axillary lymph nodes, colon, heart, kidney, liver, lung, spleen and thymus were taken from each rat for histopathological evaluation. Blood was collected for biochemical and haematological analyses. Blinded analysis was performed by an independent veterinary pathologist.

HCV NS proteins have been reported to induce tumour growth [41,42,43,44,45]. Therefore, to determine if the HCV vaccine caused any abnormalities including neoplasia at the site of injection, the ears from vaccinated mice were formalin fixed, processed into paraffin wax and tissue sections examined by light microscopy by an independent veterinary pathologist, 144 days post vaccination. All animal pathology studies were blinded to the pathologist and were only revealed after the histological analysis of the harvested tissues.

### 2.5. Interferon-γ (IFN-γ) Enzyme-Linked Immunosorbent Spot (ELISpot) Assay

IFN-γ ELISpot assay was performed to assess HCV-specific CMI in vaccinated mice. As described previously [29,30,31,37,46] panels of overlapping 11−15 mer peptides spanning the entire NS3, NS4A, NS4B, and NS5B proteins (strain K3a/650, genotype 3a) were obtained from the National Institutes for Health Bio Defense and Emerging Infectious Research Resources Repository, NIAID, National Institutes of Health. The peptides were divided into pools, each containing 29−31 individual peptides. Briefly, multiscreen-IP HTS plates (Millipore # MILMSIPS4510) were coated overnight at 4 °C with anti-mouse IFN-γ antibody (clone AN18, MabTech #3321-3-1000). Single cell suspensions of murine splenocytes, depleted of red blood cells, were stimulated with peptide pools of HCV NS3, NS4A, NS4B and NS5B at a final concentration of 4 µg/mL for 36 h at 37 °C. Secreted IFNγ was detected with anti-mouse IFNγ-biotin (clone R4-6A2, MabTech), followed by streptavidin-alkaline phosphatase (Sigma) and SigmaFast BCIP/NBT. Polyhydroxyalkanoates (PHA)-stimulated cells (5 µg/mL) were used as a positive control and splenocytes cultured in media (unstimulated) represented negative controls. Developed spots were counted automatically using an ELISpot reader (AID GmbH, Germany). The number of spots present in unstimulated splenocytes was subtracted from the number in peptide-stimulated cells to generate the net number of specific spots forming units (SFU).

### 2.6. Statistical Analysis

Statistical analysis and graphical representation of data were performed using GraphPad Prism version 6.00 for Windows (GraphPad Software, La Jolla, CA, USA). Data were presented as mean ± SEM. When comparing three or more groups, a global significance was first determined by unpaired two-tailed, non-parametric Kruskal–Wallis test with * *p* ≤ 0.05 being considered significant. Statistical analysis between groups was performed using the unpaired Mann–Whitney tests. Histopathological incidence was scored and analysed using Fisher Exact test. Values of * *p* ≤ 0.05, ** *p* ≤ 0.01, *** *p* ≤ 0.001 were considered significant. The statistical analysis was performed with the assistance of our departmental statistician. 

## 3. Results

### 3.1. Optimal Dose of DNA Vaccines Encoding HCV Antigens

**Figure 2:**
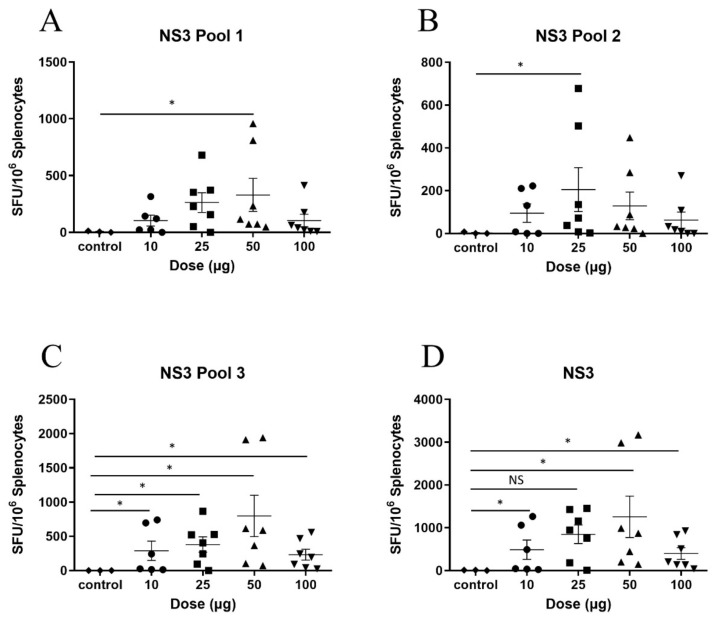
Titration of pNS3 in C57BL/6 mice. Mice were vaccinated three times at two week intervals with pNS3 and splenocytes were harvested 14 days post final vaccination. Splenocytes were re-stimulated in duplicate with overlapping peptides representing the complete HCV NS3 protein (gt3a) and IFN-γ secretion detected by ELISpot analysis. Splenocytes from vaccinated animals were re-stimulated with overlapping peptides representing (**A**) NS3 pool 1 (**B**) NS3 pool 2 (**C**) NS3 pool 3 (**D**) NS3 combined analysis. Each symbol represents an individual mouse and data are shown as mean ± SEM of SFU per 10^6^ splenocytes. Significance shown between control and dose groups. No significant differences were observed between dose groups. * *p* ≤ 0.05 (Mann–Whitney test non-parametric *t*-test).

To determine the optimal dose, the DNA vaccines were titrated. The mice were vaccinated with 10, 25, 50 or 100 µg of pNS3, pNS4B5B or 100 µg of pVAX and two weeks after the final vaccination, the HCV-specific T-cell response was measured by IFN-γ ELISpot. The mean SFU in splenocytes from mice vaccinated with 10, 25 or 50 µg pNS3 and restimulated with NS3 pool 1 peptides showed a dose-dependent increase from 104 to 263 and 330 SFU, respectively, although this was not significant. Interestingly, mice vaccinated with 100 µg of pNS3 showed a mean SFU of 104 (Figure 2A). Restimulation of the splenocytes with the NS3 Pool 2 and 3 peptides (Figure 2B,C) resulted in data consistent with that from Pool 1. Mice vaccinated with 50 µg showed the highest response (mean SFU 1258) (Figure 2D)

**Figure 3:**
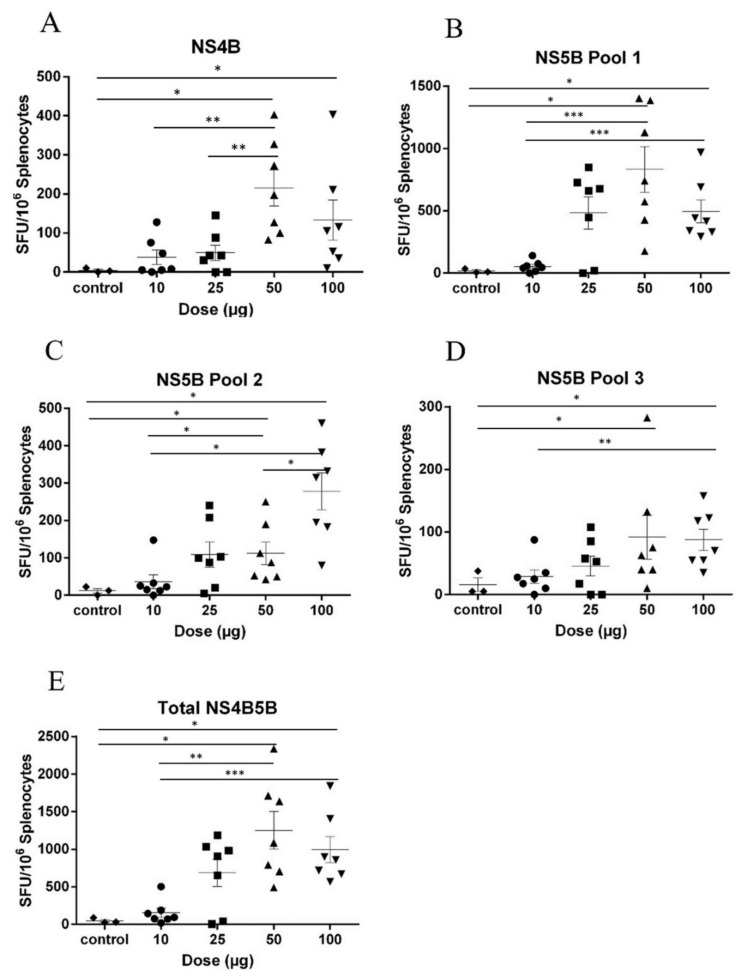
Titration of pNS4B/5B in C57BL/6. Mice were vaccinated three times at two week intervals with pNS4B/5B and splenocytes were harvested 14 days post final vaccination. Splenocytes were re-stimulated with overlapping peptides representing the HCV (**A**) NS4B (**B**) NS5B Pool 1 (**C**) NS5B Pool 2 (**D**) NS5B Pool 3. (**E**) NS4B and NS5B combined analysis. Each symbol represents an individual mouse and data are shown as mean ± SEM of SFU per 10^6^ splenocytes. Significance shown between control and dose groups. No significant differences were observed between dose groups. * *p* ≤ 0.05; ** *p* ≤ 0.01; *** *p* ≤ 0.001 (Mann–Whitney test non-parametric *t*-test).

After vaccination with pNS4B5B, the mean SFU of splenocytes restimulated with NS4B or NS5B Pool 1 peptides (Figure 3A,B) was similar to that from the pNS3 titration, as the mean SFU showed a dose-dependent increase that peaked in mice that received 50 µg (mean SFU 216 and 833, respectively) and was reduced in re-stimulated splenocytes from mice vaccinated with 100 µg (mean SFU 133 and 497, respectively). In contrast to Pool 1, the restimulation of splenocytes with NS5B peptide Pool 2 showed an increase in SFU corresponding to the escalation of the dose (Figure 3C), with higher responses in mice that received 100 µg compared to mice that received 50 µg (mean SFU 278 ) while NS5B Pool 3 showed no significant difference between the 50 µg and 100 µg groups (mean SFU 92 vs. 88) (Figure 3D). However, this did not change the overall dose–response curve for the accumulated mean SFU, as the 50 µg dose was shown to be optimum while mice vaccinated with 100 µg showed a decreased response in comparison (mean SFU 1253 v 995) (Figure 3E).

Using the titration data described above, we showed previously that vaccination with 50 µg of a multi-antigenic vaccine, pNS345B-PRF, elicited higher CMI responses than the pNS345B vaccine lacking the PRF molecule [31]. Thus, as the pNS345B-PRF vaccine is clearly superior, we then examined the toxicity of this vaccine.

### 3.2. No Long-Term Pathological Outcomes in C57BL/6 Vaccinated Mice

As it has been reported that several HCV proteins, including NS3 and NS4B, are linked to tumour development [41,42,43,44,45], we evaluated any long-term effects resulting from the HCV DNA vaccine injected via the ID route. Tissue samples were collected from the injection site 144 days post final injection, and histological studies were undertaken by an independent veterinary pathologist. 

No adverse histopathological findings related to the vaccination of mice with pNS3/4A/4B/5B-PRF or pNS3/4A/4B/5B were observed when compared to mice which received PBS (Table 1). The reported dermal inflammation, hyperkeratosis, dermal neutrophils and scab were thought to be background lesions caused by the group housing of the mice for the duration of the experiment and were not significant. No treatment-related findings were observed in mice vaccinated with pNS3/4A/4B/5B or pNS3/4A/4B/5B-PRF.

Mice were vaccinated three times at two week intervals with pNS3/4A/4B/5B-PRF, pNS3/4A/4B/5B or PBS, and 144 days post final vaccination mice were euthanised and the site of injection was assessed by blinded analysis by an independent veterinarian. The number of ears that present histopathological incidence were scored. (C57BL6, *n* = 7/group).

### 3.3. Toxicology, Haematology and Biochemical Studies in Vaccinated Rats.

Before human clinical trials can be performed, it is essential to establish that the multi-antigenic cytolytic DNA vaccine is not only efficacious, but is also safe. Consequently, the local and systemic effects of the DNA vaccines were assessed in male Sprague-Dawley rats, which were vaccinated with saline, pNS3/4A/4B/5B or pNS3/4A/4B/5B-PRF. To ensure stringency, the animals were primed with 150 μg of DNA followed by vaccination with 450 μg DNA on Days 5 and 15. This vaccine dose was 10−20-fold higher relative to proposed doses in humans, as determined by body surface area (BSA) calculations [47,48]. The rats were weighed daily after each vaccine dose. One week after the final vaccination, they were euthanized; several organs including lung, spleen, heart, liver, and kidneys were collected, weighed and examined histologically. 

**Figure 4:**
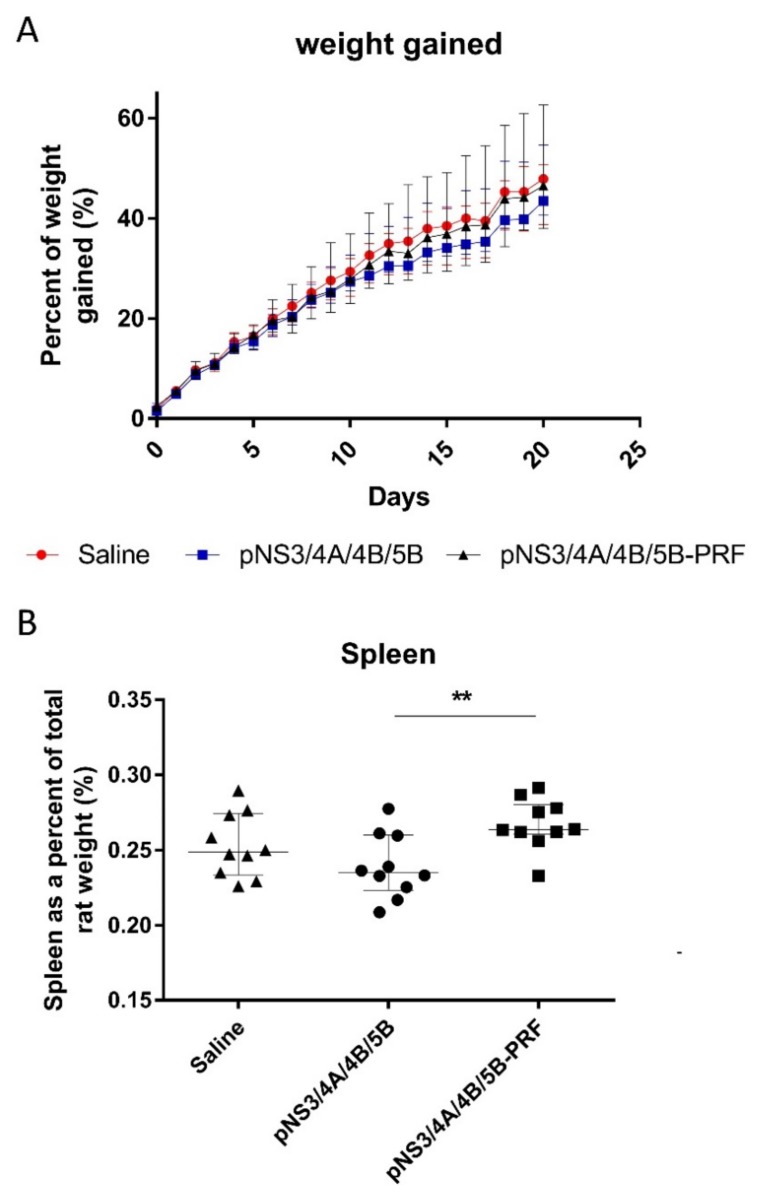
Weight of Sprague Dawley rats after vaccination. Rats were injected with 150 μg of DNA on Day 0 followed by 450 μg DNA on Days 5 and 15, then killed and necropsies performed on Day 23. Weight of (**A**) rat was measured daily and increase graphed as a percentage and (**B**) spleen as a percentage of total rat weight. Data shown as median (*n* = 10) and interquartile range. ** *p* ≤ 0.01 (Mann–Whitney test).

**Figure 5:**
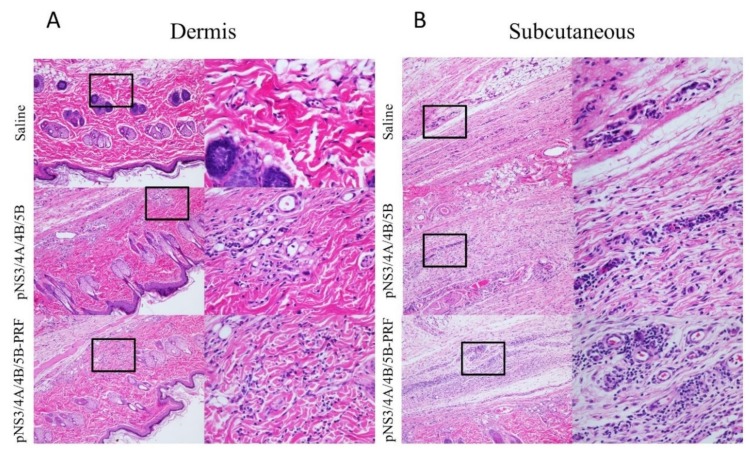
Histological representation of site of injection in Sprague Dawley rats. Histological representation of lymphocyte infiltration in the (**A**) dermis and (**B**) subcutaneous layer at the site of injection in rats vaccinated with Saline, pNS3/4A/4B/5B, and pNS3/4A/4B/5B-PRF. Images on left are 10× objective, insets on the right are 40× magnification.

No difference in the weights of animals in any of the groups was observed (Figure 4A). The weight of each individual organ viz. lung, spleen, heart, liver, and kidneys was also measured, with no differences between groups (data not shown) except the spleen (Figure 4B), as rats that received the pNS3/4A/4B/5B-PRF vaccine showed a significantly enlarged spleen (*p* = 0.0052) compared to rats that received the pNS3/4A/4B/5B vaccine. This increase in size is indicative of an inflammatory immune response and was not considered detrimental. No detrimental consequence was noted in the histological reports of the individual organs including the spleen. Histopathological changes related to treatment were detected in the skin and subcutis from the injection site of rats that received pNS3/4A/4B/5B or pNS3/4A/4B/5B-PRF compared to the animals injected with saline (Figure 5A,B) as determined by an increase in the incidence and severity of dermal inflammation, subcutis inflammation, panniculus myositis and subcutis fibrosis, (Table 2). No significant differences were noted between the two vaccinated groups. Analysis of the biochemistry and haematology results of the blood collected from the rats showed no significant changes between the cytolytic and canonical DNA vaccinated groups (data not shown). Increased inflammation in the experimental groups is thought to be a result of antigen-specific expression induced by vaccine delivery in both groups (canonical and cytolytic DNA).

These findings are not unexpected for a DNA vaccine and are not considered to be adverse. Apoptosis was observed in the thymus in almost all animals as well as occasionally in the periarteriolar sheaths of the spleen, however these are normal background changes in the rat thymus [49] and spleen, and are not considered to be of toxicological significance. Alveolar haemorrhage was observed in the lungs of some rats in all three groups. This finding is most likely to have been caused by the carbon dioxide asphyxia method of euthanasia and is not thought to be significant.

## 4. Discussion

As HCV gt3 has become increasingly common in the intravenous drug user (IDU) population, and is now the most prevalent genotype in the UK and South Asia with a high prevalence in Australia [50,51,52,53], a vaccine is urgently required.

Currently, four DNA vaccines are licensed for use in animals, while none are licensed for use in humans [54,55]. As one of these vaccines protects against West Nile Virus (WNV) infection in horses [56], this demonstrates that the mass of the vaccinated subject is not a limiting factor for DNA vaccine efficacy. This WNV DNA vaccine can also elicit NAbs in humans after a three dose course that results in similar protective levels to those in horses [55].

Furthermore, DNA vaccines have shown considerable promise in clinical trials. Trimble et al. and Kim et al. reported the use of therapeutic DNA vaccines that target human papillomavirus (HPV) 16 and 18 in cervical intraepithelial neoplasia (CIN) 2/3 patients [23,24]. Trimble et al. showed that their vaccine (VGX-300) elicited robust CMI responses that resolved persistent HPV infection and prompted regression of cervical lesions in 40% of patients compared to 14% in the placebo group, with similar responses seen in the modified-to-treat group [24]. The study performed by Kim et al. reported a phase I dose escalation study of a therapeutic vaccine (GX-188E) targeting HPV-16 and 18 in CIN3 patients. At 36 weeks after vaccination it was reported that seven out of nine patients cleared persistent HPV infection with complete regression of CIN3 lesions [23]. These studies highlight several important points for DNA vaccines: they can be efficacious in humans, function in a therapeutic manner to resolve persistent viral infection and are safe.

We have previously reported that mice vaccinated with a multi-antigenic vaccine resulted in more robust CMI compared to those vaccinated with a limited antigen vaccine and analysis of the immune responses after DNA vaccination showed that the inclusion of cytolytic PRF improved the immunogenicity of the HCV antigens [30,31]. Using a challenge model we developed to test vaccine efficacy in mice, we demonstrated that the cytolytic vaccine resulted in an accelerated lymphocyte migration to the liver compared to unvaccinated mice [30,57], a key factor in preventing chronic HCV infection [58,59,60]. Furthermore, we showed that a multi-genotype HCV vaccine could target multiple genotypes without compromising single genotype specificity [37]. To increase credibility, outbred white Landrace pigs, a recognised animal model for vaccine evaluation, were also vaccinated in a preclinical model to test the translational potential of our vaccine technology [30]. Pigs received three doses of 300 μg of either pVAX-NS3 or pVAX-NS3-PRF using a microneedle device to ensure reproducible delivery of the vaccines. Compared to the canonical vaccine, the cytolytic vaccine increased the magnitude of the initial effector cell response, which dictates the magnitude of the population of central memory T cells, important for vaccine efficacy [55].

We have previously shown that a two or three dose regimen induced robust T-cell responses in mice and pigs, and a three dose regimen is likely to be considered acceptable for clinical trials [30,31]. Mice vaccinated with high doses of DNA were reported to show reduced responses compared to those that received a lower dose [61]. Herein, a similar pattern was observed, as a higher dose resulted in reduced CMI and a dose of 50 µg was considered optimal. All animals were observed closely following vaccination but no noticeable changes in behaviour were observed and no adverse effects were reported. In the long-term toxicology study, a few mice showed some minor pathological events, but these were thought to be background lesions caused by interactions during housing and not a consequence of vaccination. Previous studies have shown that DNA vaccines do not induce significant side effects in vaccinated individuals [17,62,63] and thus are regarded as safe.

A primary concern relates to potential side effects resulting from the expression of PRF, as it is theoretically possible that autoimmunity may develop against the host animal’s PRF. However, no antibodies against PRF in animal serum were detected by Western blot analysis after cytolytic DNA vaccination [30]. Furthermore, it is considered highly unlikely that, even if anti-PRF antibodies were generated, they would have a detrimental effect, for a number of reasons: (i) the immunological synapse where host PRF is released from intracellular stores has a size exclusion which inhibits solutes such as antibodies from entering [64] (ii) the interaction of the host cell PRF at the immunological synapse occurs within seconds of being secreted and thus does not allow sufficient time for antibody interaction [35].

Although PRF-mediated cell death may raise some concerns with regulatory bodies, it is well established that many vaccine technologies cause cell death viz. live attenuated viral vaccines, electroporation and aluminum salts [65,66,67,68,69,70] but are nevertheless regarded as safe. 

A study by Lee et al. showed that a DNA vaccine, VGX-6150, which encoded NS3, NS4, and NS5 of HCV was safe and has entered phase I clinical trial (NCT02027116) [71]. Their study showed similar immune response and safety profile as reported by us, however our DNA vaccine does not require electroporation and is administered at a lower dose.

The data outlined herein and in references [30,31,37] demonstrate that the cytolytic vaccine caused no side effects in small and large animals, meeting safety standards expected of a DNA vaccine [62].

## 5. Conclusions

The aim of this study was to examine the safety of a DNA vaccine in Sprague Dawley rats and C57BL/6 mice. The multi-antigenic cytolytic vaccine was shown to be safe with no adverse events reported. Thus, this cytolytic DNA vaccine design has been demonstrated to be safe and efficacious and is a promising candidate for human trials.

## Figures and Tables

**Table 1:** Histopathological incidence at the site of injection in vaccinated C57/BL6 mice.

Group	pNS3/4A/4B/5B-PRF		pNS3/4A/4B/5B		PBS	
Ear	Left	Right	Left	Right	Left	Right
Number examined	7	7	7	7	7	7
No detectable pathology	4	3	4	1	4	1
Scab						
Minimal	0	1	0	0	0	0
Slight	1	0	0	0	0	0
Dermal Neutrophils						
Slight	1	1	0	0	0	0
Moderate	0	0	1	0	0	0
Dermal Inflammation						
Minimal	3	1	2	3	2	4
Slight	0	1	0	1	1	1
Moderate	0	0	0	2	0	1
Total	3	2	2	6	3	6
Hyperkeratosis						
Minimal	0	0	0	0	1	0
Slight	0	0	1	0	0	0

**Table 2:** Histopathological incidence at the dermal site of injection in Sprague Dawley rats.

Group	pNS3/4A/4B/5B-PRF	pNS3/4A/4B/5B	PBS
Dermal Inflammation			
Minimal	2	3	0
Slight	1	3	1
Total	3	6 *	1
Subcutis Inflammation			
Minimal	0	1	5
Slight	2	2	0
Moderate	8 **	7 **	0
Total	10 *	10 *	5
Panniculus Myositis			
Minimal	3	4 *	0
Slight	0	2	0
Total	3	6 **	0
Subcutis Fibrosis			
Minimal	1	1	0
Slight	1	5 *	0
Moderate	0	1	0
Total	2	7 **	0

Rats were vaccinated three times with pNS3/4A/4B/5B-PRF, pNS3/4A/4B/5B and PBS. Ten days after the final vaccination, rats were euthanised and the site of injection excised and analyzed. The number of rats that presented with histopathological incidence was scored. *n* = 10, *p ≤ 0.05, ** *p* ≤ 0.01 (Fisher Exact test).
